# Serum IL-33 Levels Are Associated with Liver Damage in Patients with Chronic Hepatitis C

**DOI:** 10.1155/2012/819636

**Published:** 2012-01-24

**Authors:** Juan Wang, Pingwei Zhao, Hui Guo, Xiguang Sun, Zhenyu Jiang, Lijun Xu, Junyan Feng, Junqi Niu, Yanfang Jiang

**Affiliations:** ^1^Department of Central Laboratory, The Second Part of First Hospital, Jilin University, Changchun 130032, China; ^2^Department of Hepatology, First Hospital, Jilin University, Changchun 130032, China

## Abstract

Interleukin-33 (IL-33) is associated with the development of Th2 responses. This study examined the potential role of IL-33 in the pathogenic process of chronic hepatitis C (CHC) in Chinese patients. The levels of serum IL-33 and sST2 in 154 patients with CHC, 24 with spontaneously resolved HCV (SR-HCV) infection and 20 healthy controls (HC), were analyzed by ELISA. The concentrations of serum IL-2, IFN-*γ*, TNF-*α*, IL-4, IL-6, and IL-10, HCV loads, ALT, AST, and HCV-Ab were measured. We found that the levels of serum IL-33 in CHC patients were significantly higher than those of SR-HCV and HC but decreased after treatment with interferon for 12 weeks. More importantly, the levels of serum IL-33 were correlated with the concentrations of ALT and AST in CHC patients. The levels of serum sST2, as a decoy receptor of IL-33, were significantly higher in CHC and SR-CHC patients than those in HC, and there was no correlation between the levels of serum sST2 and IL-33. The concentrations of serum IFN-*γ* and IL-6 in CHC patients were significantly lower than those of SR-HCV. These data suggest that IL-33 may be a pathogenic factor contributing to CHC-related liver injury.

## 1. Introduction

Hepatitis C virus (HCV) is one of the major causes of chronic liver disease in the world [1]. More importantly, many of those patients with chronic hepatitis C eventually develop cirrhosis and hepatocellular carcinoma [2]. Following infection with HCV, only about 15% of patients can clear the virus, while 60–80% of patients develop persistent chronic infection [3, 4]. Previous studies have revealed that the persistence of viral infection and chronic inflammation are dependent on the interaction among the virus, hepatocyte, and the host immune system [4, 5]. The viral infection and related hepatocyte injuries are known to suppress the immune system [6, 7]. Although experimental evidence suggests that antigen-specific Th1 immunity and proinflammatory cytokines play an important role in the HCV-related liver injury and clearance of viruses [8–10], the pathogenesis of chronic HCV infection has not been fully understood.

Interleukin-33 (IL-33) is one of the newly described members in the IL-1 family and can be produced by epithelial tissues and vascular endothelial cells [11]. IL-33 binds to its heterodimer receptors composed of IL-1 receptor-related protein ST2 and IL-1 receptor accessory protein (IL-1RaP) and can activate the MyD88 and NF-*κ*B-related signal pathway [11, 12]. ST2 has transmembrane form of ST2 (ST2 or ST2L) and soluble form of ST2 (sST2). ST2 is expressed on Th2 and mast cells and functions as a mediator of IL-33 bioactivities, while sST2 acts as a decoy receptor for IL-33. Biologically, IL-33 induces Th2 cell differentiation and activates mast cells, leading to Th2 cytokine production and Th2 response as well as pulmonary and mucosal Th2 inflammation [11]. Furthermore, IL-33 can antagonize the LPS-induced mortality in a model of septic shock [12], and the levels of serum IL-33 were elevated in SLE and RA patients [13]. Recently, IL-33 has been found to be an important factor of the pathogenesis of HIV infection and dengue virus infection [14, 15]. However, little is known on whether IL-33 could participate in the pathogenic process of HCV infection.

In the current study, we examined the concentrations of serum IL-33 and sST2 in patients with chronic hepatitis C (CHC), individuals with spontaneously resolved HCV infection (SR-HCV), and healthy controls (HC) to evaluate the potential role of IL-33/ST2 axis in the pathogenic process of CHC. Furthermore, we determined the concentrations of serum IL-33 before and after antivirus therapies in patients with CHC. We found that IL-33 response appeared to be an important factor of the pathogenesis of CHC, associated with the severity of liver injury in CHC patients. We discuss the implications of our findings.

## 2. Materials and Methods

### 2.1. Patients

A total of 154 patients with CHC, 24 cases with SR-HCV, were recruited sequentially at the outpatient service of First Hospital. Another 20 gender-, age- and ethnic-matched HC were recruited, and they had no historical liver diseases and no evidence of HBV, HCV, and HDV infection. Individuals with positive anti-HCV antibodies and serum HCV RNA for at least six months were diagnosed with CHC [16]. Individuals with SR-HCV were defined as those subjects who had prior HCV RNA detection (HCV-Ab^+^) but lacked HCV RNA detection for 12 weeks after enrollment in the absence of treatment [17]. Genotyping of HCV showed that 22 CHC patients had genotype 2a, 45 had genotype 1b, and 14 had unclassified genotype. Individuals with historical and current hepatitis B, D virus or HIV infection, autoimmune hepatitis, or metabolic liver disease, who had received immunosuppressive therapy or antiviral therapy during the past 12 months before entry, were excluded. All patients denied drug use or exposure to obvious hepatotoxin. Their demographic and clinical characteristics are shown in Table 1.

Fifty patients with CHC were treated subcutaneously with 500 million units of a short-acting interferon (SINOGEN, Jinan, China) every other day for 12 weeks. The concentrations of serum IL-33, sST2, HCV-RNA, ALT, and AST were measured before and after treatment. Individuals with 100-fold reduced serum virus load were defined as drug-responsive patients; otherwise, individuals were defined as drug-nonresponsive patients. Written informed consent was obtained from individual participants, and the study was approved by the First Hospital Ethical Committee of Jilin University.

Peripheral blood samples were obtained from individual subjects, and their sera were prepared and then stored at −80°C till needed. The levels of serum IL-33 were measured at the entry of individual participants, before and after treatment of patients with CHC, and the concentrations of serum IFN-*γ*, TNF-*α*, IL-2, IL-4, IL-10, and IL-6 in individual CHC patients were also determined.

### 2.2. Measurement of IL-33 and sST2 by ELISA

The concentrations of serum IL-33 and sST2 in individual patients and healthy controls were determined by ELISA using human IL-33 and sST2 ELISA kits, according to the manufacturers' instruction (Roche Diagnostics, Lewes, UK). Briefly, individual sera at 1 : 4 dilutions were subjected to ELISA analysis, and the concentrations of serum IL-33 and sST2 in individual samples were calculated, according to the standard curve established using the recombinant IL-33 and sST2 provided. The detection limitation of the IL-33 and sST2 ELISA kit was 0–16 ng/L and 1.6 ng/L, respectively.

### 2.3. Cytometric Bead Array of Serum Cytokines

The concentrations of serum cytokine levels (IFN-*γ*, TNF-*α*, IL-2, IL-4, IL-10, and IL-6) were determined by cytometric bead array (CBA) [18], according to the manufacturer's protocol (BD Biosciences, San Joes, USA) with minor modification. Briefly, 25 *μ*L of individual sera was used in duplicate for analysis, as described previously [19]. The concentrations of serum cytokines were quantified using the CellQuestPro and CBA software (Becton Dickinson) on a FACSCalibur cytometry (BD Biosciences).

### 2.4. Serologic Analysis of Hepatitis

The concentrations of serum antibodies against HCV were detected by ELISA II (Abbott Laboratories, Abbott Park, USA) [20]. The levels of serum ALT and AST were detected using a Biochemistry Automatic Analyzer (Roche Diagnostics, Branchburg, USA). The amounts of serum HCV RNA were measured by quantitative PCR assay using a luciferase quantization detection kit, following the protocols (Roche Amplicor, Basel, Switzerland). The detection limit of viral RNA was 300 copies/mL.

### 2.5. Statistical Analysis

The data are expressed as median and range unless specified. The differences between the groups were analyzed by Wilcoxon-rank sum test and chi-square test using the SPSS 14.0 software. The relationship between variables was evaluated using the Spearman rank correlation test. A two-sided *P* value <0.05 was considered statistically significant.

## 3. Results

To determine the potential role of IL-33 in the pathogenic process of CHC, a total 154 patients with CHC, 24 with SR-HCV, and 20 with HC were sequentially recruited. As expected, there was no significant difference in the distribution of age and gender among these groups of subjects, but the concentrations of serum ALT and AST in patients with CHC were significantly higher than those in the HC and those with SR-HCV (Table 1). While high levels of virus RNA were detected in CHC patients, there was no detectable viremia in both HC and SR-HCV. In addition, the anti-HCV antibody was detected in CHC patients and individuals with SR-HCV, but not in HC.

**Figure 1:**
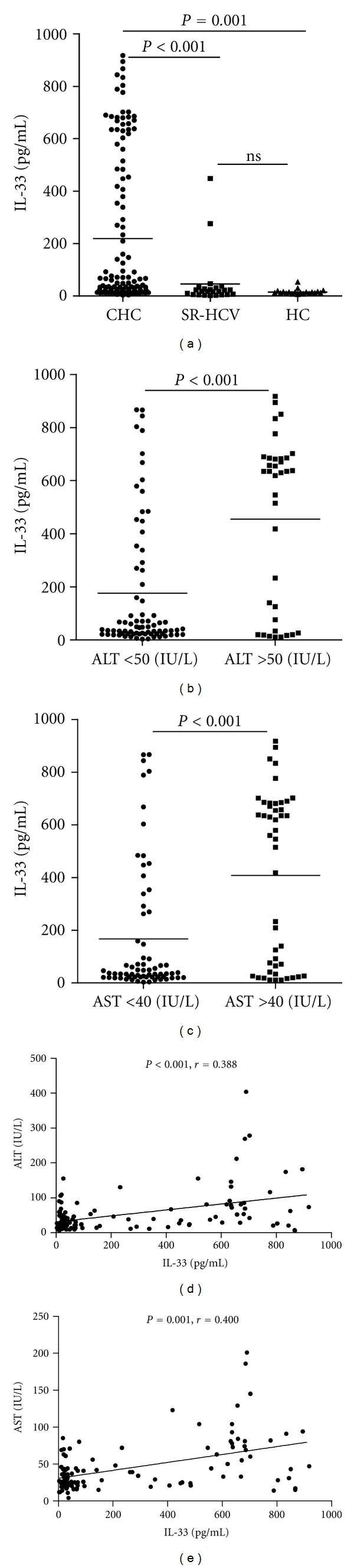
The levels of serum IL-33. The concentrations of serum IL-33 in individual CHC patients, those with SR-HCV and HC, and the levels of serum ALT and AST were determined by ELISA and automatic enzymatic assays, respectively. The potential association of the levels of serum IL-33, ALT, and AST was analyzed using the Spearman rank correlation test. Data are expressed as the mean values of individual participants from two separate experiments. The horizontal lines indicate the median values of different groups. (a) The basal levels of serum IL-33; (b) the levels of serum IL-33 in those with different levels of serum ALT; (c) the levels of serum IL-33 in those with different levels of serum AST; (d) the correlation between the levels of serum IL-33 and ALT; (e) the correlation between the levels of serum IL-33 and AST. HC: healthy controls; CHC: patients with CHC; SR-HCV: individual with spontaneously resolved HCV patients (SR-HCV).

Analysis of serum cytokines indicated that there was no significant difference in the concentrations of serum IL-33 between individuals with SR-HCV and HC, while the concentrations of serum IL-33 in patients with CHC were significantly higher than those in individuals with SR-HCV and HC (*P* < 0.001, Figure 1(a)).

**Figure 2:**
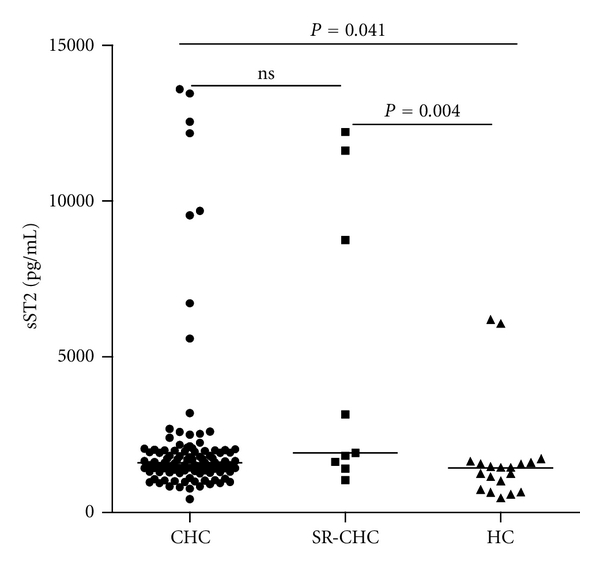
The levels of serum sST2. The concentrations of serum sST2 in individual CHC patients, those with SR-HCV and HC, were determined by ELISA. Data are expressed as the mean values of individual participants from two separate experiments. The horizontal lines indicate the median values of different groups.

**Figure 3:**
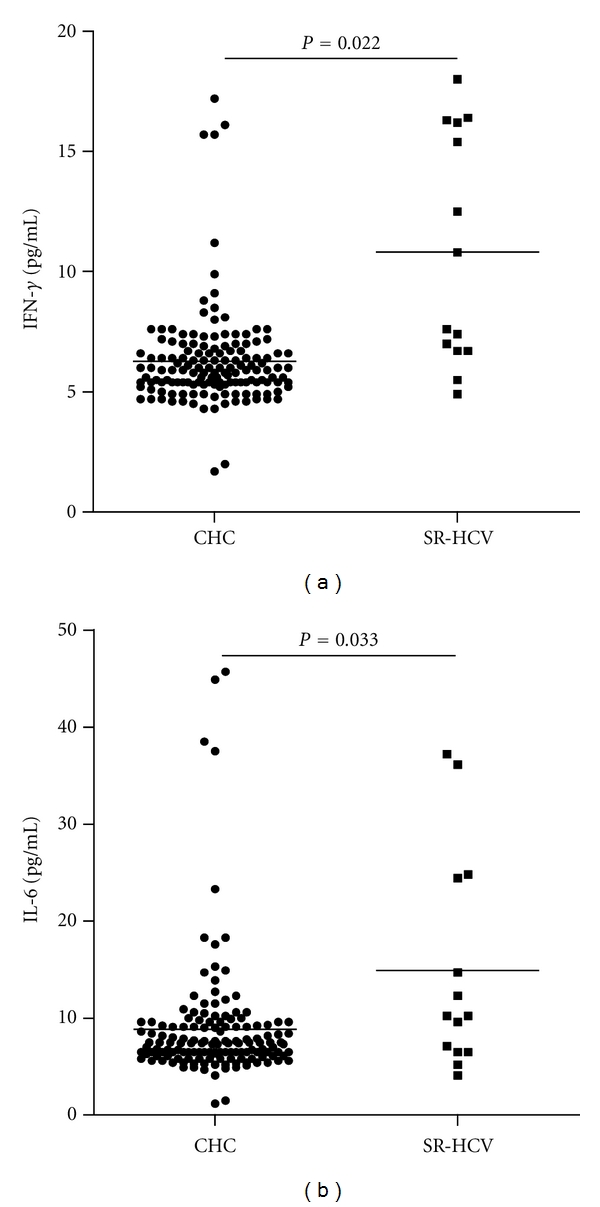
The basal levels of serum IFN-*γ* and IL-6. The concentrations of serum IFN-*γ* and IL-6 in individual participants were determined by CBA. Data are expressed as the mean values of individual samples from two separate experiments. The horizontal lines show the median. (a) The levels of serum IFN-*γ*; (b) the levels of serum IL-6.

**Figure 4:**
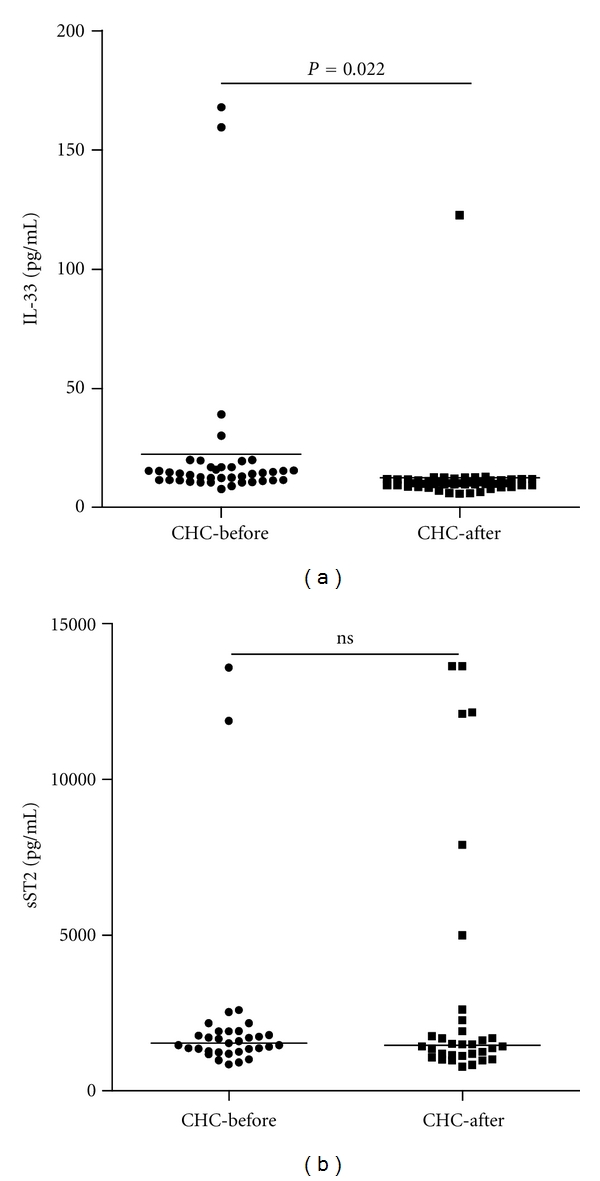
The changes in the levels of serum IL-33 and sST2 in CHC patients with IFN treatment. Data are expressed as the mean values of individual participants from two separate experiments. The horizontal lines show the median. (a) The changes in the levels of serum IL-33 in CHC patients with IFN treatment; (b) the changes in the levels of serum sST2 in CHC patients with IFN treatment.

 Furthermore, stratification of patients with CHC revealed that the concentrations of serum IL-33 in CHC patients with abnormal levels of serum ALT (>50 units/L) or AST (>40 units/L) were significantly higher than those in CHC patients with normal levels of ALT (<50 units/L) or AST (<40 units/L), respectively (*P* < 0.001, *P* < 0.001, resp., Figures 1(b) and 1(c)). The concentrations of serum IL-33 in CHC patients were correlated positively with the levels of serum ALT and AST (*r* = 0.388, *P* < 0.001; *r* = 0.400, *P* < 0.001, resp., Figures 1(d) and 1(e)). Apparently, IL-33 is a pathogenic factor, associated with the damage of the liver in CHC patients. Further analysis revealed that the levels of serum sST2 were significantly higher in CHC and SR-CHC patients than that in HC (*P* = 0.004, *P* = 0.041, resp., Figure 2), but there was no significant correlation between the levels of serum IL-33 and sST2 in those subjects (*r* = −0.050, *P* = 0.678). Moreover, the concentrations of serum IFN-*γ* and IL-6, but not TNF-*α*, IL-2, IL-10, and IL-4, in patients with CHC, were significantly lower than those in individuals with SR-HCV (Figures 3(a) and 3(b)). Following treatment of CHC patients with IFN for 12 weeks, 45 out of 50 CHC patients displayed dramatically reduced levels of serum HCV virions. Notably, treatment with IFN remarkably reduced IL-33 responses in those patients because the levels of serum IL-33 in patients with CHC after treatment with IFN were significantly lower than those before treatment. (*P* = 0.002, Figure 4(a)). However, treatment with IFN for 12 weeks did not change significantly in the levels of serum sST2 in CHC patients (*P* = 0.641, Figure 4(b)).

## 4. Discussions

IL-33 is a multifunctional cytokine involved in various disease conditions [21–23]. IL-33, through the receptor complex composed of ST2 and IL-1RaP, can activate the MAP kinase and NF-*κ*B signal pathways and promote Th2 response and cytokine production [11]. Indeed, intranasal administration of IL-33 triggered an immediate allergic response in the airway, and endogenous IL-33 contributes to airway inflammation [24]. A recent study revealed that the levels of serum IL-33 were elevated in SLE and RA patients and correlated with the levels of serum ESR and CRP, two inflammation markers, indicating that IL-33 may participate in the acute-phase response of SLE [13]. In addition, IL-33 can protect against septic shock by enhancing neutrophils infiltration at the site of inflammation [12]. Other studies suggest that IL-33 participates in the pathogenic process of acute hepatitis induced by Con-A [25, 26], and IL-33 overexpression is associated with the development of HBV/HCV-related liver fibrosis [27]. To investigate the role of IL-33 in the pathogenic process of CHC, we determined the levels of serum IL-33 and sST2 in 154 CHC patients. We found that the levels of serum IL-33 in CHC patients were significantly higher than in those with SR-HCV and HC. Furthermore, the levels of serum IL-33 in CHC patients with abnormal concentrations of ALT or AST were significantly higher than in those with normal levels of ALT and AST in this population. In addition, treatment with IFN to inhibit the replication of HCV dramatically decreased the levels of serum IL-33 in CHC patients. More importantly, the concentrations of serum IL-33 were correlated positively with the levels of serum ALT and AST in CHC patients. Given that abnormal levels of ALT and AST are indicative of abnormal liver function and injuries, our data suggest that IL-33 may be a pathogenic factor of the pathogenic process of CHC in Chinese patients. Therefore, if the levels of serum IL-33 are also correlated with pathogenic degrees of the liver in CHC patients, the levels of serum IL-33 may be used as a new biomarker for the diagnosis of liver damages in CHC patients. Furthermore, we found that the levels of serum sST2 were significantly higher in CHC and SR-CHC patients than those in HC, but were not correlated with the levels of IL-33 in patients. Treatment with IFN for 12 weeks did not significantly change the levels of serum sST2. A previous study has shown that the levels of serum sST2 in patients with acute liver failure are higher than in those with chronic liver failure and healthy controls [28]. It is possible that high levels of serum sST2 are an early biomarker of liver injury, while high levels of serum IL-33 may be associated with the development and progression of liver fibrosis and damage [27]. We are interested in further examining the mechanisms underlying the action of IL-33/sST2 axis in HCV-related liver injury.

We found that the levels of serum IFN-*γ* and IL-6 in CHC patients were significantly lower than those in SR-HCV, supporting the notion that proinflammatory cytokines, such as IFN-*γ* and IL-6, are not only important factors for the clearance of infected HCV, but also for liver injury [29–31]. The lower levels of IFN-*γ* and IL-6 in CHC patients were unlikely to have come from the antagonization of IL-33-induced Th2 responses in those patients, because we failed to detect significant difference in the levels of serum IL-4 and IL-10 between those CHC patients and SR-HCV and HC. Given that IL-6 is a critical factor of the functional development of Th17 cells [32] and that IFN-*γ* is an effector of Th1 response [33], the lower levels of serum IFN-*γ* and IL-6 in those CHC patients indicated continual viral replication and pathogenic progression. Although IL-33 has been shown to promote IFN-*γ* production by invariant NKT and NK cells [34], IL-33 may, through an unknown pathway, downregulate the functional development of HCV-related Th1 response and inhibit IFN-*γ* production. However, the precise mechanisms remain to be further investigated.

 In conclusion, our data indicate, for the first time, that the concentrations of serum IL-33 are significantly higher in those with SR-HCV and HC and are significantly correlated with the levels of serum ALT and AST, suggesting that IL-33 may be a pathogenic factor of HCV-related liver injury in CHC patients. We recognized that the current study has limitations, including no source for IL-33 and no histopathological examination of liver tissues. Although more detailed studies are necessary to determine the role and mechanisms of IL-33 in regulating the pathogenic process of CHC, our novel findings may provide new insights into understanding the pathogenesis of CHC.

## Figures and Tables

**Table 1:** Demographic characteristics and clinical features of participants.

Parameters	HCV	SR-HCV	Healthy controls
Number	154	24	20
Age (years)	47 (28–65)	52 (31–57)	47 (42–54)
Sex (M/F)	110/44	15/9	14/6
Viraemia (log_10_ copies/mL)	6.0 (1.3–7.6)	NA	NA
ALT (U/L)	25 (5–420)*	21 (7–87)	14 (5–26)
AST (U/L)	31 (4–226)*	17 (6–57)	12 (8–32)
Anti-HCV	Positive	Positive	Negative

Normal values (NA): ALT ≤ 50 IU/L; AST ≤ 40 IU/L; HCV RNA ≤ 300 copies/mL; *P < 0.05 versus SR-HCV or HC. Data were expressed as median and range.
